# Does mental health coaching improve efficacy of transcranial magnetic stimulation for major depression? A pilot randomized controlled trial and benchmarking study

**DOI:** 10.1016/j.jad.2026.121366

**Published:** 2026-02-09

**Authors:** Benjamin M. Rosenberg, Nora M. Barnes-Horowitz, Doan Ngo, Jung Woon Park, Ossanna H. Amran, Aleeza West, Jiani Li, Chiana Yang, Kelly Y. Cai, Thomas E. Valles, Cole Matthews, Isabelle Lanser, Jill M. Newby, Michael Millard, Andrew F. Leuchter, Michelle G. Craske

**Affiliations:** a Neuroscience Department, Pomona College, Claremont, CA, USA; b Department of Psychology and Neuroscience, University of Colorado Boulder, Boulder, CO, USA; c Department of Psychiatry and Biobehavioral Sciences, University of California, Los Angeles (UCLA), Los Angeles, CA, USA; d TMS Clinical and Research Service, Neuromodulation Division, Semel Institute for Neuroscience and Human Behavior at UCLA, Los Angeles, CA, USA; e Division of Biology and Medicine, Brown University, Providence, RI, USA; f School of Psychology & Marriage and Family Therapy, Fuller Theological Seminary, Pasadena, CA, USA; g Department of Psychology, University of California, Los Angeles (UCLA), Los Angeles, CA, USA; h Department of Psychology, University of Southern California, Los Angeles, CA, USA; i College of Osteopathic Medicine of the Pacific, Western University of Health Sciences, Pomona, CA, USA; j Dana-Farber/Boston Children’s Cancer and Blood Disorders Center, Boston, MA, USA; k School of Psychology, Faculty of Science, University of New South Wales, Sydney, NSW, 2052, Australia; l Black Dog Institute, Hospital Road, Randwick, NSW, 2031, Australia; m Clinical Research Unit for Anxiety and Depression, St Vincent’s Hospital, 390 Victoria Street, Darlinghurst, Sydney, New South Wales, 2010, Australia

**Keywords:** Depression, Transcranial magnetic stimulation, Mental health coaching, Cognitive behavioral therapy, Clinical trial

## Abstract

Major Depressive Disorder (MDD) is common and burdensome. Repetitive Transcranial Magnetic Stimulation (rTMS) is recommended for individuals who do not respond to first-line treatments. Coach-supported digital mental health programs are scalable strategies for delivering therapeutic content. This randomized controlled trial tested whether digital mental health programs bolster rTMS for MDD.

*N* = 36 depressed adults completed a six-week course of rTMS. Of this group, *n* = 18 were randomized to digital cognitive behavioral therapy (iCBT with Coaching), and n = 18 were randomized to digital narrative stories of hope (iNarratives with Coaching). Multilevel models tested group differences in treatment outcomes over time. Principal outcomes focused on the Hamilton Rating Scale for Depression. Exploratory analyses compared outcomes versus a Benchmarking Sample of *N* = 29 patients who received rTMS without coaching.

There was a main effect of time (*p* < .001), indicating an overall reduction in depression symptoms during the trial. Contrary to hypotheses, there was no Group × Time interaction (*p* = .662), and groups did not differ on clinical response rates (*p* = .654). Secondary analyses found that the iNarratives group showed greater improvement in positive emotion and functional impairment, whereas the iCBT group showed less dropout from the coaching intervention. Exploratory analyses found a Group × Time interaction (*p* = .039), potentially suggesting that participants in iCBT or iNarratives showed steeper symptom reduction versus the Benchmarking Sample.

The coach-supported iCBT and iNarratives approaches are comparable as adjuncts to rTMS for depression and may yield lower depression scores versus rTMS alone. iNarratives effects appeared specific to increases in positive emotions and decreases in functional impairment.

## 1. Introduction

Major Depressive Disorder (MDD) is a highly burdensome and costly condition (Greenberg et al., 2023; König et al., 2020). MDD affects nearly 4–5% of the global population at a given time and 10–30% of the global population at some point during their lifetime (Marx et al., 2023), and global rates of MDD have been increasing (Goodwin et al., 2022; Liu et al., 2020; Moreno-Agostino et al., 2021). First-line treatments for MDD include psychotherapy or antidepressant medications (Solomon et al., 2019), and their combination is considered the optimal standard of care (Cuijpers et al., 2020; Karyotaki et al., 2016). Nonetheless, a large proportion of individuals do not achieve substantial improvement from first-line treatments (Cuijpers et al., 2021a, 2021b).

Repetitive Transcranial Magnetic Stimulation (rTMS) is a safe and effective intervention recommended for MDD patients who do not respond to first-line treatments (Trapp et al., 2025). rTMS is thought to directly target and manipulate neurocircuitry that is implicated in MDD. For example, standard rTMS treatments target the left dorsolateral prefrontal cortex because of its association with systems responsible for emotion regulation and mood, such as the subgenual anterior cingulate cortex and amygdala (Oathes et al., 2023). Rates of MDD remission from rTMS are approximately 20–30%, although there is some variability depending on the measure used (Leuchter et al., 2023; Sackeim et al., 2020). Furthermore, over half of those who respond to rTMS are estimated to relapse after one year (Senova et al., 2019). Thus, while rTMS exhibits substantial clinical utility, improvements are needed to enhance treatment response and durability.

As standard rTMS treatments do not involve the acquisition of psychological skills, it has been suggested that combining rTMS with psychotherapy could engage separate mechanisms or additively target common mechanisms (e.g., top-down mechanisms of emotion regulation) to amplify treatment response or reduce relapse (Markowitz et al., 2021; Sathappan et al., 2019; Tatti et al., 2022; Tsagaris et al., 2016). Indeed, preliminary studies have shown that combining rTMS and psychotherapy for MDD is feasible (Vedeniapin et al., 2010) and may improve treatment efficacy (Donse et al., 2018; Russo et al., 2018). However, there are several barriers to integrating psychotherapy into rTMS clinics during routine clinical care, including limited availability of licensed clinical psychologists trained to deliver evidence-based psychotherapy (Kazdin and Blase, 2011). Additionally, rTMS is not typically integrated into psychotherapy clinics, as it requires substantial technological and clinical infrastructure (Einstein and Leuchter, 2024).

Digital mental health programs, such as internet-based Cognitive Behavioral Therapy (iCBT), provide a scalable and accessible alternative to clinician-delivered psychotherapy (Ramos et al., 2024; Schueller and Torous, 2020) and have demonstrated effectiveness for MDD (Hedman-Lagerlöf et al., 2023; Karyotaki et al., 2021). Given their utility and accessibility, digital mental health programs may be especially well-suited for combination with other treatments (Newby et al., 2021) such as rTMS. To our knowledge, only one study has been conducted looking at combining digital mental health with rTMS for MDD – that study used an unguided iCBT program and did not find an added benefit compared with rTMS alone (Adu et al., 2023). However, digital mental health programs are more effective when supported by trained mental health coaches, a strategy that can maintain accessibility of the intervention while ensuring strong fidelity to therapeutic content (Cohen and Schueller, 2023; Rosenberg et al., 2022). It remains unknown whether a coach-guided digital mental health program may enhance treatment response during rTMS for MDD, and whether specific content (e.g., CBT skills) provides an added benefit over and above non-specific factors associated with coach support.

In this pilot randomized controlled trial (RCT), we compared a well-validated iCBT with coaching program versus a novel digital intervention with coaching involving narrative stories of mental health recovery during rTMS for MDD. The narrative program was a digital delivery of psychoeducation and stories of hope. Narrative coaching was modeled after supportive psychotherapy, which is designed to facilitate affective expression without an overt emphasis on skills acquisition or homework practice (Markowitz, 2022a) and has shown to be effective for MDD (Markowitz, 2022b). Considering that supportive psychotherapy has smaller effect sizes than CBT for MDD (Cuijpers et al., 2024), we hypothesized that rTMS patients who received iCBT with Coaching would show greater symptom reduction compared to the rTMS patients who received iNarratives with Coaching. In an exploratory aim, considering that approaches grounded in either CBT or supportive therapy are more effective than non-intervention (e.g., waitlist- or placebo-control) (Cuijpers et al., 2023, 2024), we compared the RCT Sample to a Benchmarking Sample of patients who received only standard-of-care rTMS treatment for MDD in the same clinic. We hypothesized that participants receiving either iCBT or iNarratives with Coaching would show greater improvement compared to the rTMS Benchmarking Sample.

## 2. Methods

This study involved a pilot RCT comparing two strategies for mental health coaching as an adjunct to rTMS treatment for MDD. All participants received rTMS treatment. The comparison between iCBT with Coaching and iNarratives with Coaching groups was pre-registered on ClinicalTrials.gov (NCT05988619).^1^ This trial did not receive external funding. Upon completion of the study, a Benchmarking Sample was created to compare these interventions to standard-of-care rTMS outcomes (see below).

### 2.1. Study participants

This study was approved by an Institutional Review Board (IRB#19–000581) at the University of California, Los Angeles (UCLA). All patients were recruited through the UCLA TMS Clinical and Research Service and provided written informed consent. Eligibility for the study was determined by certified interviewers using the Mini-International Neuropsychiatric Interview (MINI) (Sheehan et al., 1998) and the 17-item Hamilton Rating Scale for Depression (HDRS) (Hamilton, 1986). Eligibility for the study was based on the standard inclusion criteria employed by the UCLA TMS Clinical and Research Service: 1) diagnosis of current MDD based on the MINI, 2) evidence of prior depression treatment without demonstrated effectiveness (i.e., at least two antidepressant medications, at least one course of psychotherapy); 3) right-handedness^2^; 4) between 18 and 75 years of age; 5) willingness to undergo 10-Hz rTMS treatment at the outset of treatment. Participants were excluded from the study based on standard procedures in the clinic, including 1) any TMS contraindications (e.g., metal implants, pregnancy, psychosis), and 2) comorbidities that may interfere with treatment response (e.g., autism spectrum disorders, bipolar disorder, substance use dependence). Participants were additionally excluded if they reported current participation in cognitive behavioral therapy.

During an initial visit prior to the beginning of rTMS, all eligible patients were offered the opportunity to receive information about the study. All interested patients were contacted by the study team to provide more information and undergo informed consent (*N* = 100).

### 2.2. Treatment

#### 2.2.1. rTMS protocol

All participants received rTMS treatment for depression according to standard clinical protocols in the UCLA TMS Clinical and Research Service. Prior to the first treatment, resting motor threshold (RMT) was determined for each participant by identifying the minimum stimulus intensity corresponding with ≥50% overt motor response in the right abductor pollicis brevis muscles. rTMS was administered five days per week over a six-week period for a total of at least 30 sessions, starting with 3000 pulses 10 Hz rTMS administered to the left dorsolateral prefrontal cortex targeted with the Beam F3 method (Beam et al., 2009). The TMS train duration was 40 s and the inter-train interval was 11 s. Stimulation intensity was increased to 120% RMT as tolerated over the first five treatments. Following the 30-session treatment period, six additional sessions were offered on a tapered schedule (3–2–1 per week). Parameters were adjusted using a measurement-based care paradigm previously described in which subjects who had difficulty tolerating the initial protocol or demonstrated limited improvement after 10 treatments could undergo protocol adjustments^3^ to optimize response and tolerability (Citrenbaum et al., 2023; Fitzgerald et al., 2006; Chu et al., 2023; Lee et al., 2020; Leuchter et al., 2023; Mirman et al., 2022; Tadayonnejad et al., 2022, 2023). Participants were additionally permitted to extend beyond the initial treatment phase in accordance with clinician recommendations, typically involving an additional 10 sessions paid out-of-pocket. rTMS devices included MagPro X100 (Magventure), Magstim Horizon (Magstim), Magstim Super Rapid2 (Magstim), or Neurostar (Neuronetics) devices.

#### 2.2.2. Digital mental health programs

##### 2.2.2.1. iCBT with coaching.

iCBT was delivered using the *This Way Up*^4^ Anxiety and Depression Program, which has demonstrated effectiveness in the treatment of depression and anxiety across a variety of clinical settings (Mahoney et al., 2021; Newby et al., 2013, 2014). The program is separated into six lessons: 1) Psychoeducation; 2) Thought Monitoring/Activity Planning; 3) Cognitive Restructuring; 4) Exposure; 5) Advanced Exposure; 6) Relapse Prevention. Individual lessons are self-paced and designed to take no longer than 60 min each. iCBT content is presented in the form of an illustrated story, wherein two fictional characters use cognitive behavioral techniques to effectively respond to depression and anxiety symptoms. Participants were permitted to access iCBT materials at a time of their choosing. Following completion of each lesson, participants were instructed to download a document including a summary of the material and practical homework exercises to reinforce the iCBT content. Participants were encouraged to practice their lesson homework before their next coaching session. Coaching sessions were conducted once per week via videoconferencing, with sessions scheduled separately from TMS treatment visits based on the participant’s availability.

##### 2.2.2.2. iNarratives with coaching.

The iNarratives with Coaching condition comprised six videos selected from *Stories of the Mind*, a series of mental health videos created and shared by the Public Broadcasting Service (PBS) in the USA.^5^ The six topics involved firsthand stories of maintaining mental wellbeing, overcoming symptoms of depression^6^ or anxiety, building a supportive community, coping with physical illness, regulating sleep, and discussing emerging technologies in mental health. Participants were permitted to access iNarratives videos at a time of their choosing and were instructed to watch one video prior to each corresponding coaching session. In contrast to iCBT with Coaching, and consistent with approaches in supportive psychotherapy (Markowitz, 2022a), participants in this group were not instructed to download any documents or complete any homework exercises between coaching sessions. Coaching sessions were conducted once per week via videoconferencing, with sessions scheduled separately from TMS treatment visits based on the participant’s availability.

#### 2.2.3. Coach training and supervision

Coaches for the study (authors JWP, OHA, AW, JL, CY, KYC) were first trained and certified to support an iCBT intervention through the UCLA Screening & Treatment for Anxiety & Depression program (described in Rosenberg et al., 2022; Wolitzky-Taylor et al., 2023). In that program, coaches were trained in iCBT content, core process skills (e.g., nonverbal skills, open-ended questioning, reflection statements), and other cornerstone topics (e.g., ethics, cultural humility). During coaching sessions, coaches reviewed homework with participants and addressed strategies for implementing skills.

Study coaches had no prior familiarity or training regarding the iNarratives with Coaching program developed for this study. Training to support this program mirrored the iCBT with Coaching program, including education regarding *Stories of the Mind* video content and application of core process skills to support the narratives. Coaches additionally underwent didactic trainings regarding supportive psychotherapy (e.g., refraining from “change talk”) and followed content checklists to organize coaching sessions (see Supplement). During coaching sessions, coaches reviewed video content with participants and encouraged participants to relate the content to their own lives.

Throughout the study, coaches received ongoing training and supervision in weekly 60-minute meetings led by graduate student and postdoctoral clinicians (authors BMR, NMBH, and IL) and supervised by a licensed clinical psychologist (author MGC). Study training and supervision sessions focused on 1) maintaining fidelity to the study protocols for each group (see below), 2) enhancing knowledge and facility with core process skills during targeted role play exercises, and 3) facilitating peer-to-peer feedback.

Coaching sessions were not recorded to protect patient confidentiality. Following coaching sessions, coaches completed checklists to self-rate adherence to the standardized procedures for coaching in the iCBT or iNarratives groups (see Supplement).

### 2.3. Randomization and blinding

Simple randomization was conducted to determine assignment to either iCBT with Coaching (*n* = 20) or iNarratives with Coaching (n = 20). All clinicians in the UCLA TMS Clinical and Research Service, including those responsible for conducting clinical assessments, were blinded to group assignment. A separate identification number was created linking participants to the intervention received (managed by author BMR). Neither participants nor coaches were blinded during the RCT.

### 2.4. Measures

All measures were administered at baseline, mid-treatment, and post-treatment.

#### 2.4.1. Principal outcome: HDRS

MDD symptom severity was measured using the interviewer-administered 17-item version of the Hamilton Rating Scale for Depression (HDRS) (Hamilton, 1986). Each item represents a symptom domain relevant to depression (e.g., depressed mood, behavioral engagement) based on the past week. All items include a minimum score of 0 (absent) and a maximum score of 2–4, with higher scores indicating greater symptom severity. The HDRS is considered a valid and sensitive measure of depression symptoms (Carrozzino et al., 2020) and is commonly used in depression treatment trials (Leuchter et al., 2023).

#### 2.4.2. Secondary outcomes

Secondary measures were digitized and administered online using Research Electronic Data Capture (REDCap).

##### 2.4.2.1. Inventory of Depression Symptomatology.

MDD symptom severity was measured using the self-report 30-item Inventory of Depression Symptomatology (IDS) (Rush et al., 1996). The IDS is considered a valid and sensitive measure of depression symptoms (Rush et al., 1996) and is commonly used in depression treatment trials (Leuchter et al., 2023). Each item of the IDS is rated from 0 to 3 based on the past week, with higher scores indicating greater symptom severity or frequency.

##### 2.4.2.2. Dimensional Anhedonia Rating Scale.

Anhedonia symptom severity was measured using the self-report 26-item Dimensional Anhedonia Rating Scale (DARS) (Rizvi et al., 2015). The DARS is considered a valid and reliable measure of anhedonia symptoms common to MDD (Rizvi et al., 2015). Each item of the DARS is rated from 1 to 5 based on how the participant would experience pleasurable activities “right now” according to four categories: hobbies, food/drink, social activities, and sensory experiences, with lower scores indicating greater anhedonia symptom severity.

##### 2.4.2.3. Temporal Experience of Pleasure Scale.

Trait disposition in experiences of pleasure was measured using the self-report 18-item Temporal Experience of Pleasure Scale (TEPS) (Gard et al., 2006). The TEPS is considered a valid and reliable measure of anticipatory and consummatory pleasure and is inversely correlated with anhedonia symptoms common to MDD (Gard et al., 2006). Each item of the TEPS is rated based on “how true the statement is for you in general” on a Likert scale from 1 to 6 (1=“very false for me,” 6=“very true for me”), with higher scores indicating greater anticipatory or consummatory pleasure.

##### 2.4.2.4. Sheehan Disability Scale.

Disability and functional impairment were measured using the 3-item self-report Sheehan Disability Scale (SDS) (Sheehan et al., 1996). The SDS is considered a valid and reliable measure of functional impairment among psychiatric populations and is sensitive to treatment effects (Sheehan and Sheehan, 2008). Each item of the SDS is rated on a Likert scale from 0 to 10 (0=“not at all”, 10 = “extremely”) based on the extent to which symptoms have disrupted an individual’s life according to three categories: work/school, social life, and family life.

### 2.5. Data availability

Requests for deidentified data should be directed to the corresponding author.

### 2.6. Analyses

Modified intent-to-treat analyses were conducted using linear multilevel modeling in Stata 18.0 using the *mixed* function. All models included random effects of the intercept and fixed slopes for each participant. Group was included as a categorical variable in all analyses. Time was included as a continuous variable in primary analyses, with secondary analyses including Time as a categorical variable to evaluate pairwise Group comparisons. Age (continuous) and Gender (categorical) were included in all analyses as covariates.

#### 2.6.1. RCT sample

##### 2.6.1.1. Principal analyses.

HDRS was the primary outcome variable. Of the *N* = 36 participants who received coaching, *n* = 1 participant from the iCBT with Coaching condition was excluded from analysis due to participation in a co-occurring study using a personalized TMS stimulation frequency. An additional *n* = 3 subjects did not complete the HDRS at any timepoint and were therefore dropped from principal analyses, yielding a final sample of *n* = 32 participants.

We computed the main effect of Time and the Group × Time interaction in predicting depression symptoms. Response to treatment was defined by a ≥ 50% reduction on the HDRS from baseline to post-treatment (Leuchter et al., 2023). Group differences in response rates were evaluated using a chi-square test of independence. The number of completed coaching sessions and number of TMS treatments administered during the treatment period (maximum 30) were included as additional covariates in these models.

##### 2.6.1.2. Secondary analyses.

Secondary analyses focused on self-reported measures of depression and anhedonia symptoms (IDS, DARS, TEPS) and overall functional impairment (SDS). As some participants had missing data, analyses included data for *n* = 35 (IDS), *n* = 30 (DARS, TEPS), or *n* = 29 (SDS) participants.

We computed the main effect of Time and the Group × Time interaction in predicting self-reported symptoms. Number of completed coaching sessions and number of TMS treatments administered during the treatment period (maximum 30) were included as additional covariates in these analyses.

#### 2.6.2. Exploratory comparison to benchmarking sample

We generated a Benchmarking Sample from a database of patients treated by the UCLA TMS Clinical and Research Service without an adjunctive coaching program. Criteria for the Benchmarking Sample were: 1) provided consent for their data to be used in future research, 2) began receiving rTMS treatment through the UCLA TMS Clinic during the study period (March 2021 to December 2023), 3) received treatment specifically for MDD, 4) had at least one timepoint of data for the clinician-rated HDRS, 5) completed at least five rTMS treatment sessions, 6) did not participate in a co-occurring study using personalized stimulation frequencies for each patient.

*N* = 29 patients met criteria for inclusion in the Benchmarking Sample. A subset of these patients (*n* = 7/29) were offered and declined the opportunity to participate in the present study (see Supplement for analyses removing these patients). Participants in the Benchmarking Sample were similar to the RCT Sample on Age, Gender, and baseline HDRS scores (see Table 1).

HDRS was the primary outcome variable. We first computed the main effect of Time in predicting depression symptoms within the Benchmarking Sample. We then included Group as a two-level factor (RCT Sample, Benchmarking Sample) to compute a Group × Time interaction in predicting depression symptoms. Considering the Benchmarking Sample completed no coaching sessions, number of completed coaching sessions was not included as a covariate. Number of TMS treatments administered during the treatment period (maximum 30) was included as an additional covariate in this analysis.

## 3. Results

### 3.1. RCT sample

#### 3.1.1. Participant characteristics

The RCT Sample consisted of *N* = 35 participants (17 female) ages 21–74 years (M = 45.8, SD = 14.38) who completed rTMS with either adjunctive iCBT with Coaching or iNarratives with Coaching. Groups did not differ by age (t(33) = −0.456, *p* = .652) or Gender (χ^2(1)^ = 2.33, *p* = .127). The majority of study participants (*n* = 30/35; *n* = 14/17 iCBT, *n* = 16/18 iNarratives) endorsed current daily use of at least one psychotropic medication. Participant demographics are summarized in Table 1.

#### 3.1.2. Treatment dose and dropout

**Fig. 1.**
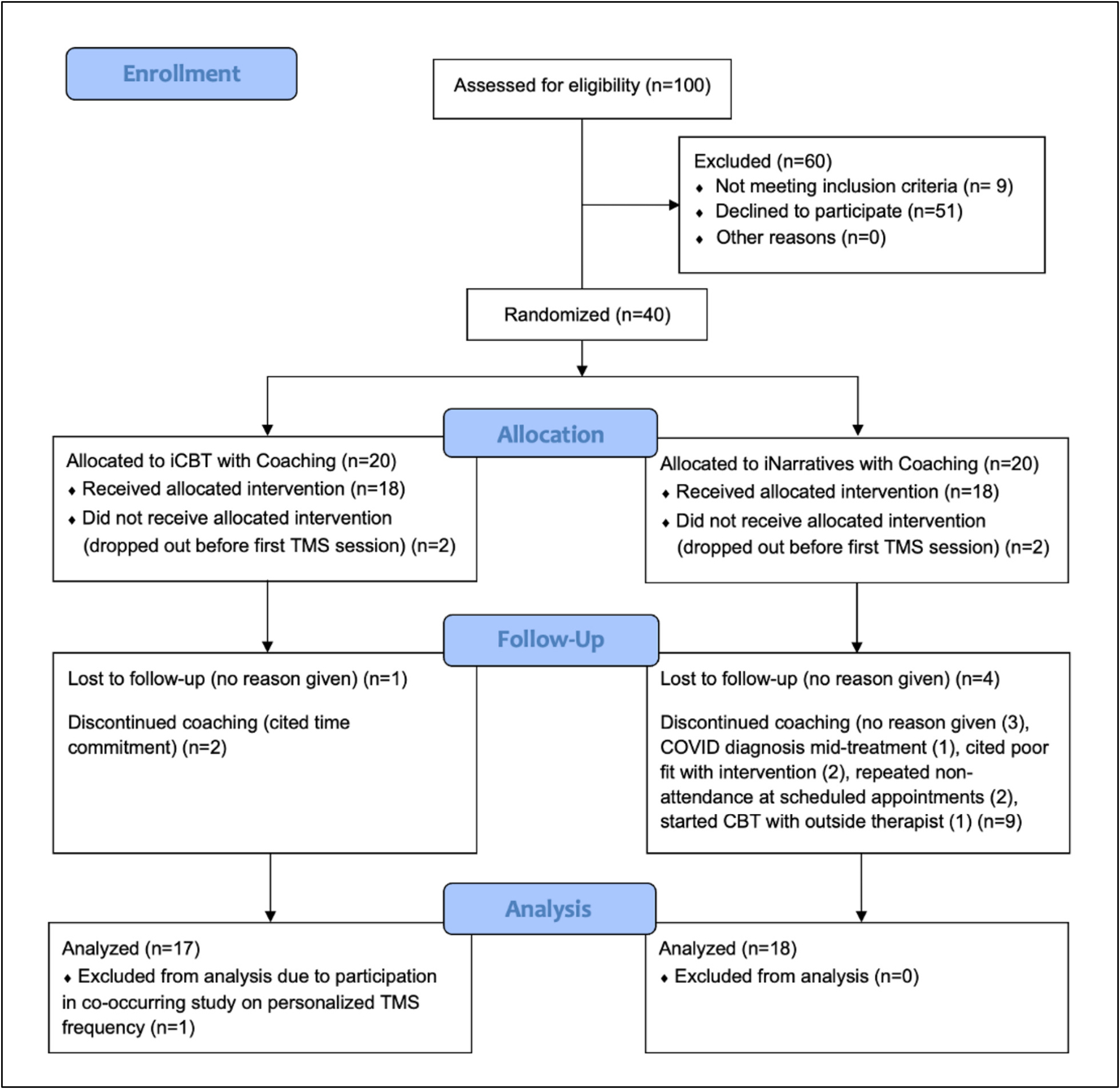
CONSORT diagram for the RCT.

There was a significant Group difference in dropout from the coaching programs (χ^2(1)^ = 8.34, *p* = .004) and number of completed coaching sessions (t(33) = 2.899, *p* = .007), such that iCBT with Coaching participants were more likely to remain in the coaching program versus the iNarratives with Coaching group (see Fig. 1 for CONSORT diagram).^7^ Among participants who attended at least one coaching session, *n* = 7 (*n* = 2 iCBT, *n* = 5 iNarratives) discontinued coaching after one coaching session. There was not a significant group difference in dropout from rTMS or number of completed rTMS sessions (*p*’s > 0.339). Treatment dose and dropout for the interventions are summarized in Table 2.

### 3.2. RCT sample: reduction in depression symptoms

#### 3.2.1. Primary outcome (HDRS) during treatment

**Fig. 2.**
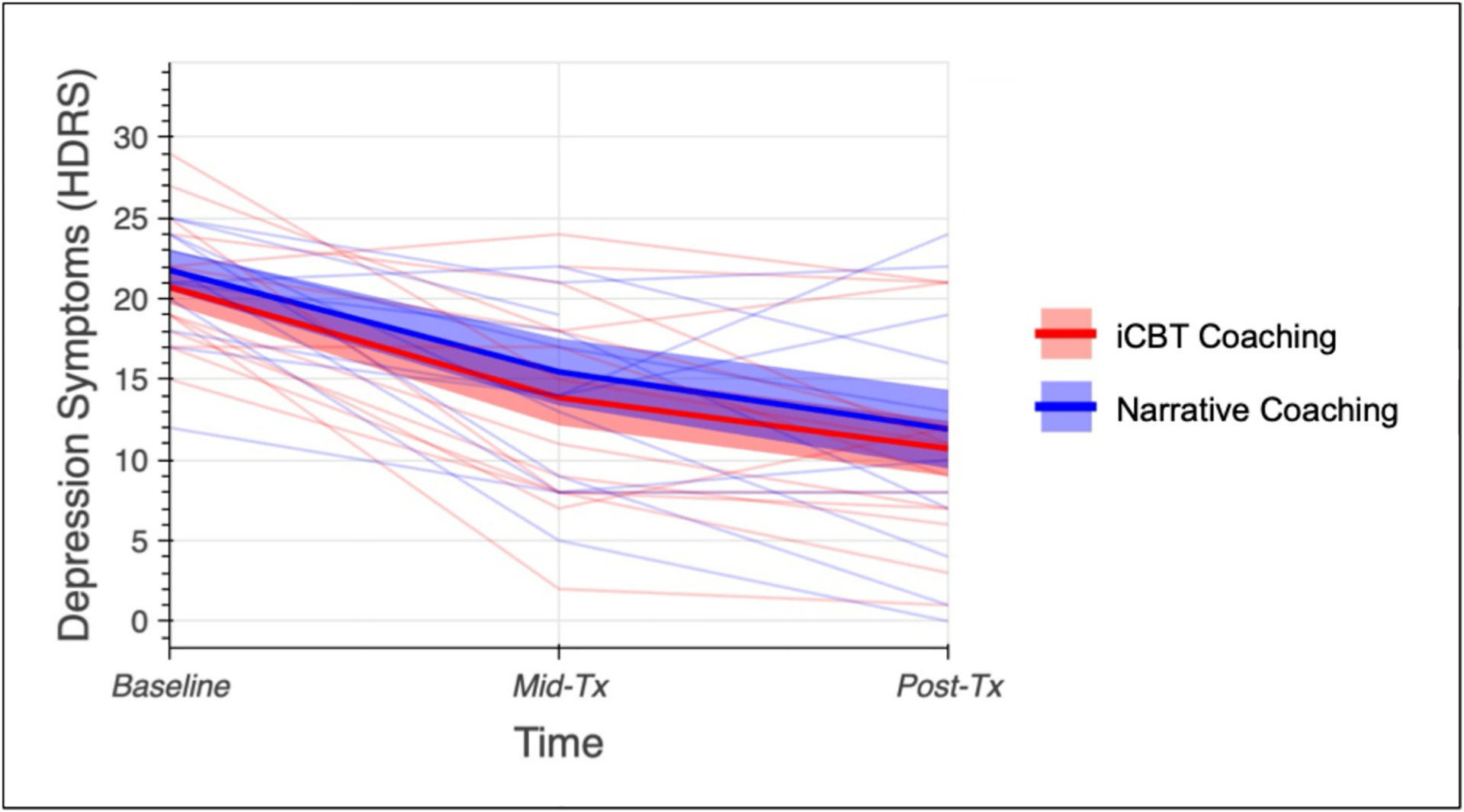
iCBT with Coaching and iNarratives with Coaching groups both showed a significant decrease in symptoms on the HDRS during treatment. Neither group outperformed the other on this measure.

There was a significant main effect of Time, such that HDRS scores decreased from baseline to post-treatment (b = −4.96, 95% CI: [−6.16, −3.75], f = 1.03, Z = −8.07, *p* < .001; Fig. 2). The Group × Time interaction was not significant (b = 0.54, 95% CI: [−1.87, 2.94], f = 0.07, χ^2 (1)^ = 0.19, *p* = .662; Fig. 2). Nor did analyses of pairwise effects show a significant group difference at mid-treatment (Z = −0.45, *p* = .653) or post-treatment (Z = −0.13, *p* = .897). Simple effects of Time were significant within iCBT with Coaching (Z = −7.05, *p* < .001) and within iNarratives with Coaching (Z = −4.58, *p* < .001).

Among those with baseline HDRS data, *n* = 12/30 individuals (40.0%) responded to the intervention (iCBT n = 7/16, 43.8%; iNarratives n = 5/14, 35.7%). There was not a significant effect of Group on the distribution of response rates (χ^2(1)^ = 0.20, *p* = .654).

#### 3.2.2. Secondary outcomes during treatment

Group differences on self-report measures are summarized in Table 3.

##### 3.2.2.1. IDS (baseline, mid-treatment, post-treatment).

There was a significant main effect of Time predicting IDS scores (b = −7.25, 95% CI: [−9.05, −5.44], f = 0.930, Z = −7.86, *p* < .001), such that participants showed a decrease in self-reported depression symptoms during treatment. There was not a significant Group × Time interaction (b = −0.26, 95% CI: [−3.87, 3.35], f = 0.017, χ^2(1)^ = 0.02, *p* = .888).

##### 3.2.2.2. DARS (baseline, mid-treatment, post-treatment, six-month, nine-month).

There was a significant main effect of Time predicting DARS scores (b = 5.27, 95% CI: [1.45, 9.08], f = 0.434, 2.71, *p* = .007), such that participants showed a decrease in self-reported anhedonia symptoms during treatment. There was not a significant Group × Time interaction predicting DARS scores (b = 3.62, 95% CI: [−4.39, 11.64], f = 0.143, χ^2(1)^ = 0.79, *p* = .375).

##### 3.2.2.3. TEPS (baseline, mid-treatment, post-treatment, six-month, nine-month).

**Fig. 3.**
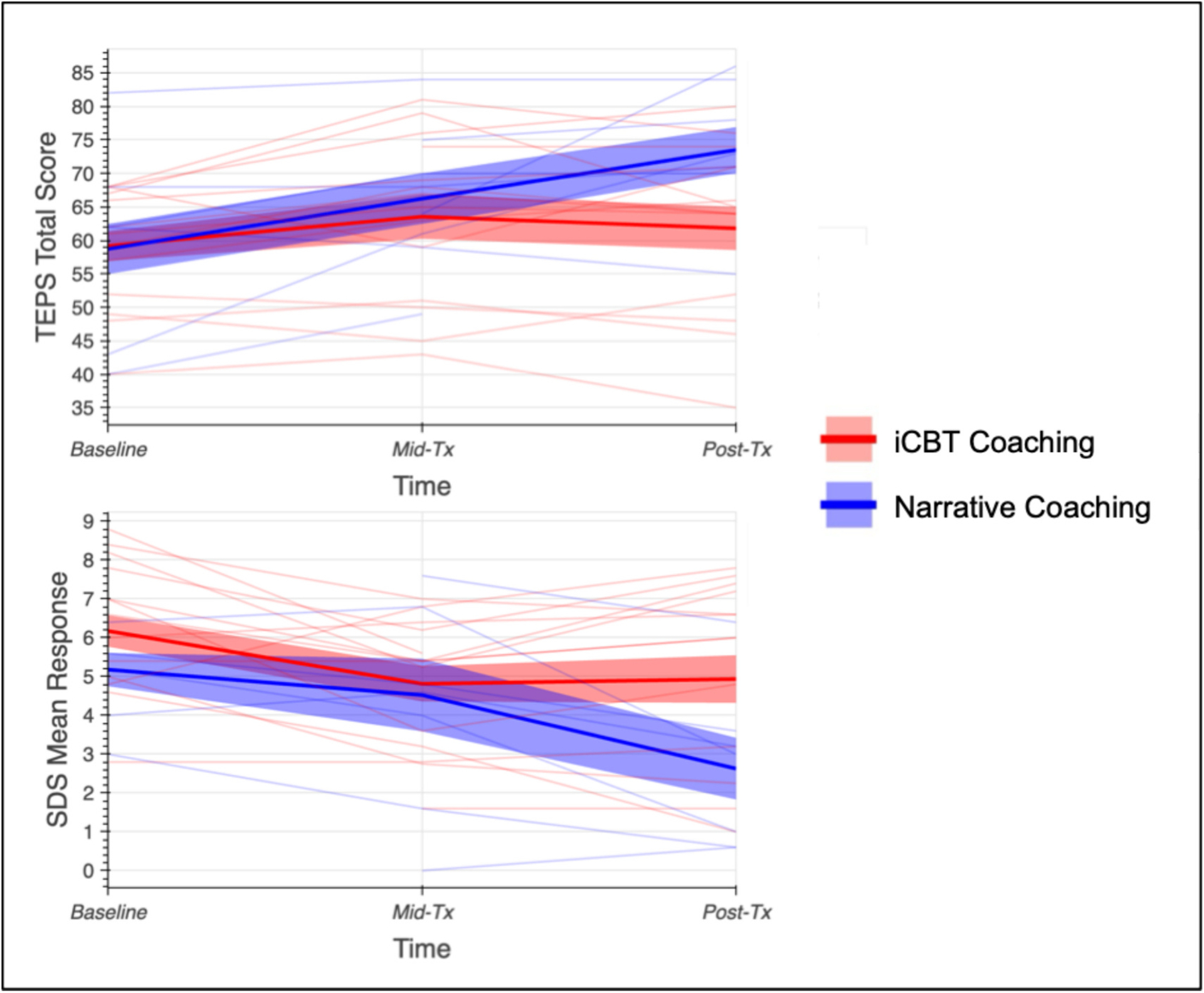
On self-report measures, participants in iNarratives with Coaching showed a significantly greater decrease in functional impairment compared with iCBT with Coaching. At the end of treatment, participants in iNarratives with Coaching showed greater self-reported pleasure and lower functional impairment compared with iCBT with Coaching.

There was a significant main effect of Time predicting TEPS scores (b = 1.94, 95% CI: [0.26, 3.61], f = 0.319, Z = 2.27, *p* = .023), such that participants showed an increase in self-reported positive emotions during treatment. There was a marginally significant Group × Time interaction predicting TEPS scores (b = 3.34, 95% CI: [−0.04, 6.72], f = 0.272, χ^2(1)^ = 3.75, *p* = .053), such that participants in iNarratives with Coaching reported a marginally greater increase in pleasure compared with the participants in iCBT with Coaching during treatment. Pairwise effects showed a significant difference (iNarratives > iCBT) at Post-Treatment (Z = 2.72, *p* = .007; Fig. 3) (all other *p*’s > 0.181).

##### 3.2.2.4. SDS (baseline, mid-treatment, post-treatment, six-month, nine-month).

There was a significant main effect of Time predicting SDS scores (b = −0.58, 95% CI: [−0.95, −0.21], f = 0.456, Z = −3.06, *p* = .002), such that participants showed a decrease in self-reported functional impairment during treatment. There was a significant Group × Time interaction predicting SDS scores (b = −0.77, 95% CI: [−1.53, −0.01], f = 0.336, χ^2(1)^ = 3.93, *p* = .047), such that participants in iNarratives with Coaching showed a greater decrease in functional impairment over time compared with the participants in iCBT with Coaching. Pairwise effects showed a significant difference (iNarratives < iCBT) at Post-Treatment (Z = −3.24, *p* = .001; Fig. 3) (all other *p*’s > 0.160).

### 3.3. Benchmarking sample

#### 3.3.1. Participant characteristics

The Benchmarking Sample consisted of *N* = 29 participants (15 female, 13 male, 1 other) ages 18–75 years (M = 41.1, SD = 15.73) who completed rTMS only. There were no significant differences between the RCT Sample and Benchmarking Sample on Age (t(62) = −1.25, *p* = .217), Gender (χ^2(2)^ = 1.38, *p* = .501), baseline HDRS scores (t(55) = −0.54, *p* = .588), or number of TMS sessions (t(62) = −1.33, *p* = .187). Patient demographics are summarized in Table 1.

#### 3.3.2. Primary outcome (HDRS) during treatment

**Fig. 4.**
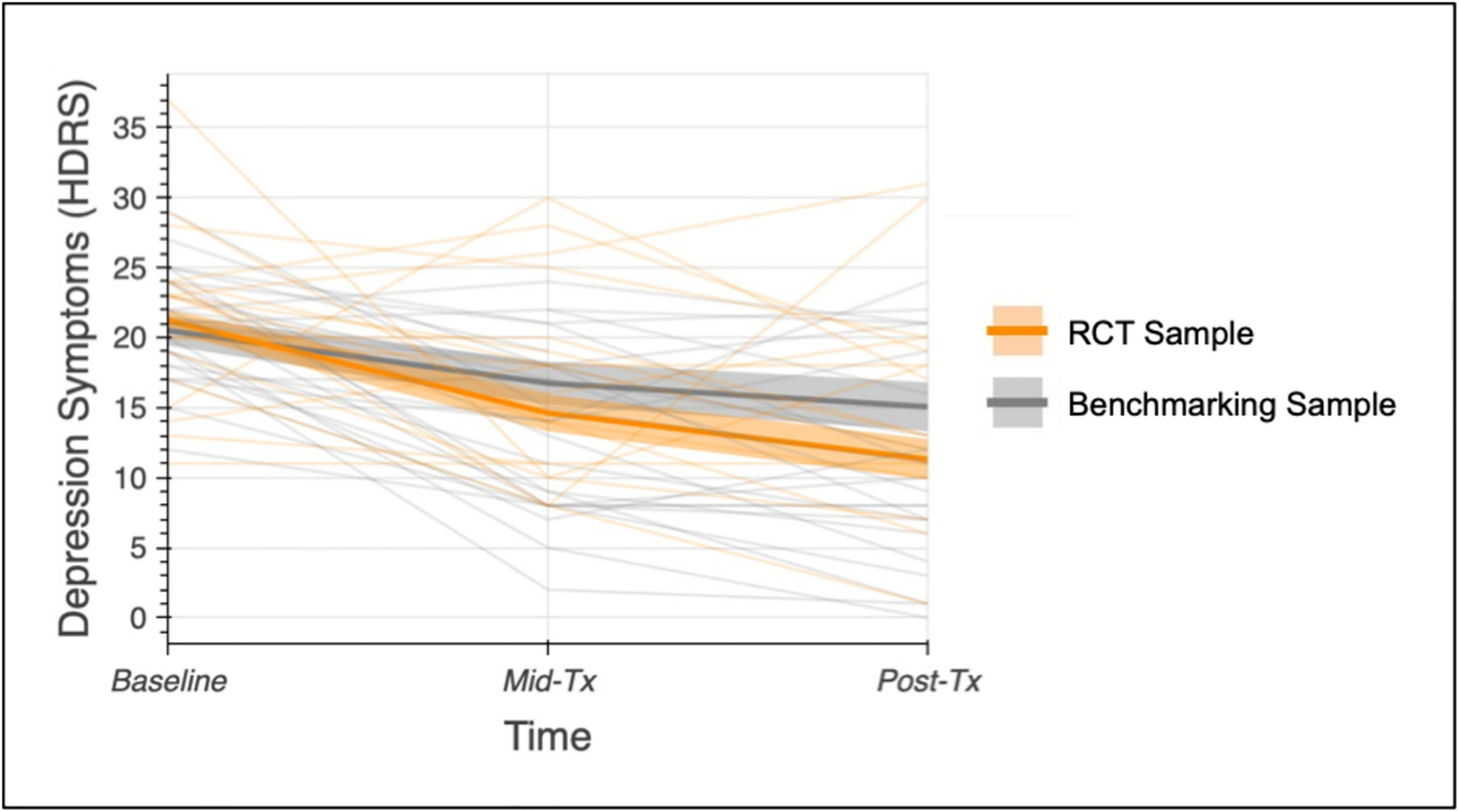
Patients in both the RCT Sample and the Benchmarking Sample showed a significant decrease in depression symptoms during treatment. The RCT Sample showed a significantly steeper decrease in depression symptoms compared to the Benchmarking Sample.

Within the Benchmarking Sample, there was a significant main effect of Time, such that individuals showed a decrease in HDRS symptoms during the treatment period (b = −2.88, 95% CI: [−4.55, −1.21], f = 0.464, Z = −3.38, *p* = .001; Fig. 4). Of the N = 29 patients, *n* = 7 (24.1%) responded to the intervention.

#### 3.3.3. Comparing RCT versus benchmarking sample

##### 3.3.3.1. Group differences on the HDRS.

There was a significant Group × Time interaction (b = −2.15, 95% CI: [−4.20, −0.10], f = 0.189, χ^2(1)^ = 4.24, *p* = .039; Fig. 4), such that participants in the RCT Sample showed a steeper decrease in HDRS symptoms during treatment period compared to the Benchmarking Sample. There were no significant pairwise difference (all *p*’s > 0.185).

##### 3.3.3.2. Response rates.

Among those with baseline HDRS data, there was not a significant difference in the distribution of clinical response (χ^2(2)^ = 1.70, *p* = .192), such that participants in the RCT Sample (*n* = 12/30, 40%) and Benchmarking Sample (n = 7/29, 24.1%) showed similar response rates.

## 4. Discussion

This pilot randomized controlled trial was designed to compare the efficacy of adding coach-supported digital mental health programs to rTMS treatment for treatment-resistant MDD. The two programs compared a) targeting specific cognitive behavioral skills, versus b) stories of hope and supportive counseling. Contrary to our hypotheses, we did not find evidence that iCBT with Coaching outperformed iNarratives with Coaching during treatment. In direct contrast with hypotheses, we found preliminary evidence suggesting that iNarratives with Coaching led to greater improvements in positive emotions and functional impairment than iCBT with Coaching. However, coaching dropout was significantly larger in the iNarratives than the iCBT with Coaching group. Consistent with our exploratory hypotheses, patients who received either coaching intervention appeared to show greater treatment efficacy compared with a Benchmarking Sample of patients who received standard-of-care rTMS (i.e., without coaching). Overall, this study provides preliminary support for the utility of coach-supported digital mental health programs as an adjunct to standard-of-care rTMS treatment for MDD. Results additionally point to the need for future research to disentangle the relative advantages of individual coaching programs.

### 4.1. Principal treatment outcomes

Although iCBT with Coaching, iNarratives with Coaching, and standard-of-care rTMS were all associated with symptom reduction during treatment, with 32.2%^8^ of patients achieving clinically significant improvement in depression across the groups, patients who received either of the coach-supported digital mental health programs showed greater symptom reduction on the primary outcome measure compared with the Benchmarking Sample. Various types of psychotherapeutic interventions for MDD (without rTMS) have shown comparable effectiveness (Barth et al., 2016; Cuijpers et al., 2008, 2021a, 2021b), and it is possible that interpersonal support from coaches may have facilitated non-specific treatment effects that ultimately yielded similar patient outcomes (Cuijpers et al., 2012; Mulder et al., 2017). Indeed, evidence suggests that unguided iCBT, which lacks non-specific support components of coach-guided digital mental health programs, does not enhance rTMS treatment of MDD (Adu et al., 2023), suggesting that coach support is a critical element of the interventions. Pending replication, results from this study suggest that coach-supported digital mental health programs may thus enhance rTMS treatments for MDD.

### 4.2. Study dropout

Despite the demonstrated efficacy of digital mental health programs for treatment of MDD, dropout remains one of the most challenging issues in the field, particularly when the program is administered without coach support (Richards and Richardson, 2012). Importantly, this study sought to mitigate treatment dropout by applying a coach-supported design, which enhances treatment compliance and retention (Furukawa et al., 2021; Mohr et al., 2011; Moshe et al., 2021; Richards and Richardson, 2012) and is associated with greater treatment efficacy and effectiveness (Karyotaki et al., 2021; Linardon et al., 2019; Mohr et al., 2019).

Results from this study suggest that iCBT with Coaching was associated with significantly less dropout than iNarratives with Coaching. There are several potential explanations for this result. First, whereas the iCBT program has previously demonstrated efficacy across a variety of clinical settings, the iNarratives program has not been tested prior to this study. Considering that the majority of patients in iNarratives with Coaching continued receiving rTMS treatments (i.e., there were no significant differences in dropout from rTMS), it is possible that the iNarratives program was less acceptable compared with the iCBT program. Indeed, among those who discontinued the iNarratives program, *n* = 2 specifically reported fit with the coaching program as part of their reasoning and *n* = 1 reported pursuing cognitive behavioral therapy with an external therapist. Alternatively, as this was a single-blind RCT with preregistered hypotheses, it is possible that coaches’ expectations about the iNarratives program may have influenced their support in ways that reduced patient engagement in this condition. Although supplemental analyses did not support this interpretation, as there were no differences in treatment credibility between the groups after the first coaching session, it is nonetheless possible that the coaches’ pre-existing training in the iCBT intervention enhanced their competence in this program compared with the novel iNarratives design. Furthermore, despite the difference in coaching dropout rates, the groups did not differ on principal treatment outcomes and response rates were similar. An alternative possibility is that participants may have discontinued the iNarratives with Coaching program specifically because their symptoms were not reducing, whereas the more structured iCBT with Coaching program retained participants even when symptoms remained elevated.

### 4.3. Secondary (self-reported) treatment outcomes

Contrary to our hypotheses, participants in iNarratives with Coaching, compared with iCBT with Coaching, showed greater increases in pleasure and greater reductions in functional impairment. There are several potential explanations for these findings. First, whereas iCBT with Coaching was largely designed to reduce negative affect symptoms common to depression and anxiety disorders (Newby et al., 2013, 2014), these strategies are often not accompanied by similar increases in positive affect (Sandman and Craske, 2022). It is possible that the more general support and validation offered by iNarratives with Coaching was particularly effective at enhancing therapeutic alliance, a mediator of change during psychotherapy (Baier et al., 2020), which in turn may have contributed to increases in positive affect and improvements in functioning. Second, although the iCBT intervention has previously been implemented among individuals across a range of depression severity, most RCTs exclude individuals with the highest severity of symptoms (e.g., suicidality) (Newby et al., 2021). In contrast, the present sample represents a population of chronically and severely depressed patients undergoing a demanding and time-intensive course of TMS treatment. It is therefore possible that patients in this study showed particular benefits from the less demanding and simplified protocol in the iNarratives program. Third, although group differences were not evident on the HDRS, self-report and clinician-administered measures are known to show discrepant results (e.g., response or remission rates) during rTMS for MDD. It is therefore recommended that rTMS clinicians administer multiple rating instruments to ensure comprehensive measurement of treatment outcomes (Leuchter et al., 2023). Finally, the secondary outcomes were administered online outside of treatment visits and therefore had a greater proportion of missing or incomplete data compared with the clinician-administered HDRS. Although multi-level modeling is sensitive to missing data, it is possible that model estimates within iNarratives with Coaching were biased by the high dropout rates (i.e., those who completed the program responses were a non-random sample) and therefore less reliable. Similarly, considering the group difference in study dropout, selective retention in iNarratives with Coaching may have inflated estimates of secondary outcomes. Future work is needed to further evaluate acceptability versus efficacy of these interventions.

### 4.4. Limitations & future directions

The results from the present study should be interpreted with caution in light of several limitations. First, the sample sizes were small, and data were missing. Future work should seek to replicate and build upon the present study to more robustly demonstrate whether coach-supported digital mental health programs augment rTMS treatment for MDD. For example, it is possible that a larger sample size would have yielded a statistically significant difference in response rates between the RCT Sample (40%) and Benchmarking Sample (24.1%). Second, as the study groups were composed primarily of White and not Hispanic or Latinx participants, additional research is needed to test these interventions across more diverse samples. Third, although the Benchmarking Sample did not differ from the RCT Sample on baseline symptom severity, Age, and Gender, it is nonetheless possible that the RCT Sample was qualitatively different from the Benchmarking Sample. For example, willingness or motivation to participate in the RCT, or knowledge that one’s treatment progress is being measured in an RCT, may be uniquely predictive of treatment success over and above other intervention components, such as the coach-supported digital mental health program. Of note, a subset of *n* = 7 individuals in the Benchmarking Sample specifically declined to participate in this study, and supplemental analyses omitting these individuals yielded only a marginally significant Group × Time effect. Waitlist controls are needed to provide a more rigorous comparison between those who receive a coach-supported digital intervention versus no additional intervention during rTMS treatment. These designs may have their own limitations (e.g., lack of placebo-control), but would nonetheless provide further evidence supporting coach-supported digital mental health programs as adjunctive treatments. Fourth, alternative intervention designs should be considered to enhance the benefit of adding coach-supported digital interventions to TMS. For example, it could be beneficial to sequence the intervention components to reduce treatment demands (e.g., an initial course of rTMS followed by the coach-supported digital intervention). Finally, it is important to consider the high rates of dropout from the study. Although nearly all participants completed a full course of rTMS for MDD, several participants discontinued the coaching component, particularly in iNarratives with Coaching. Future coach-supported digital mental health programs may be optimized to enhance rTMS treatment efficacy while maximizing patient retention.

## 5. Conclusion

This RCT and clinical benchmarking study was the first to test the combination of a coach-guided digital mental health program with rTMS in the treatment of MDD. Although the iCBT and iNarratives approaches did not differ on principal outcomes, the iNarratives approach appeared to show superior performance on secondary outcomes, whereas the iCBT approach showed less patient dropout. Comparisons to a clinical benchmarking sample provide preliminary support for the efficacy of coach-guided DMHPs as an adjunct to rTMS for major depression. Additional research with larger sample sizes is needed to further substantiate this conclusion.

## Supplementary Material

1

## Figures and Tables

**Table 1:** Participant characteristics in the study.

	iCBT Coaching (*n* = 18)		Supportive Coaching (n = 18)		Benchmarking Sample (*n* = 29)	
Age	**M = 45.67**	**SD = 15.24**	**M = 46.89**	**SD = 14.05**	**M = 41.10**	**SD = 15.73**
Gender	**n**	**Percent**	**n**	**Percent**	**n**	**Percent**
Female	6	33.33%	10	55.55%	15	51.72%
Male	12	66.67%	8	44.44%	13	44.83%
Other	0	0.00%	0	0.00%	1	3.45%
Race	**n**	**Percent**	**n**	**Percent**	**n**	**Percent**
African American/Black	1	5.56%	0	0.00%	0	0.00%
American Indian/Alaska Native	0	0.00%	0	0.00%	0	0.00%
Asian American	0	0.00%	0	0.00%	2	6.90%
Caucasian	16	88.89%	14	77.78%	16	41.38%
Mixed	0	0.00%	2	11.11%	0	0.00%
Native Hawaiian/other Pacific Islander	0	0.00%	0	0.00%	0	0.00%
Not reported	1	5.56%	2	11.11%	11	37.93%
Other	0	0.00%	0	0.00%	0	0.00%
Ethnicity	**n**	**Percent**	**n**	**Percent**	**n**	**Percent**
Hispanic or Latino	1	5.56%	3	16.67%	1	3.45%
Not Hispanic or Latino	17	94.44%	15	83.33%	18	62.07%
Not reported	0	0.00%	0	0.00%	10	34.48%
Baseline HDRS score	**M = 20.41**	**SD = 3.97**	**M = 22.00**	**SD = 4.51**	**M = 20.48**	**SD = 5.73**

**Table 2:** Group differences in treatment dropout and treatment dose among the RCT Sample.

	Treatment dropout			
	rTMS		Coaching	
	iCBT with coaching	iNarratives with coaching	iCBT with coaching	iNarratives with coaching
Number of Participants	0 (0.0%)	1 (5.6%)	1 (5.9%)	9 (50.0%)
Statistic	χ^2(1)^ = 0.97		χ^2(1)^ = 8.34	
Significance	*p* = .324		*p* = .004	
	Treatment dose			
	rTMS		Coaching	
	iCBT with coaching	iNarratives with coaching	iCBT with coaching	iNarratives with coaching
Mean number of sessions during treatment	30.00	29.50	5.71	3.89
Statistic	t(33) = 0.971		t(33) = 2.899	
Significance	*p* = .339		*p* = .007	

**Table 3:** Group × Time and pairwise effects comparing iCBT Coaching vs Supportive Coaching on self-report measures. No effects favored the iCBT Coaching group. Significant effects favoring the Supportive Coaching group are highlighted in blue.

Self-Report Measure	Interaction	Pairwise Comparisons		
	Group × Time	Baseline	Mid-Tx	Post-Tx
IDS	N.S.	N.S.	N.S.	N.S.
DARS	N.S.	N.S.	N.S.	N.S.
TEPS	χ^2(1)^=3.75, *p*=.053	N.S.	N.S.	*p*=.007
SDS	χ^2(1)^=3.93, *p*=.047	N.S.	N.S.	*p*=.001

## Footnotes

1 The registration on ClinicalTrials.gov uses the term “Psychoeducation” to refer to the iNarratives with Coaching group.

2 After the beginning of the study period, research has since found no difference in the efficacy of rTMS administered to the left dorsolateral prefrontal cortex in the treatment of MDD among right- versus left-handed individuals (Fitzgerald et al., 2021). Nonetheless, to maintain protocol consistency, and considering the potential impacts of handedness on electroencephalography assessments conducted during this study, right-handedness remained as an inclusion criterion for this study.

3 Protocol adjustments are based on established methods in the clinic (Lee et al., 2020). Briefly, these involved an augmentation strategy (adding a three-minute theta-burst protocol before the main protocol) or responding to cases of rTMS intolerance (changing from 10 Hz to 1 Hz stimulation at right dorsolateral prefrontal cortex).

4
https://thiswayup.org.au/

5 https://www.pbs.org/show/stories-mind/.

6 During the course of the study, *n* = 2 participants reported discomfort during the video *Overcoming Depression*, which includes a story from an individual who endorsed a history of suicidal ideation. In response to participant feedback, this video was replaced with the video titled *A Place to Belong*.

7 Patients were permitted to continue receiving rTMS treatments even if they discontinued the coaching portion of the study. In such cases, these patients continued to complete the clinician-rated HDRS administered by the UCLA TMS Clinic (primary outcome measure) but did not complete self-report questionnaires (secondary outcome measures).

8 This estimate aligns with a recent study analyzing retrospective data from the UCLA TMS Clinical and Research Service, which found that 33% of patients (*N* = 708 adults) showed a clinically significant response (defined as ≥50% reduction in symptoms on the HDRS, as in the present study) (Leuchter et al., 2023).

## Appendix A. Supplementary data

Supplementary data to this article can be found online at https://doi.org/10.1016/j.jad.2026.121366.
