# Supplementary material for: Does mental health coaching improve efficacy of transcranial magnetic stimulation for major depression? A pilot randomized controlled trial and benchmarking study
Source: J Affect Disord. Author manuscript; Available in PMC 2026 Jul 5. (PMC13332852; doi:10.1016/j.jad.2026.121366)
Supplement: 1 [file NIHMS2191120-supplement-1.docx]

**SUPPLEMENT**

**Treatment Credibility Ratings**

Following the first coaching session, participants provided self-reported ratings of treatment credibility. The scale included four items, each rated on a Likert scale from 0-8 (0=”Not At All,” 8=“Very”), with higher scores indicating greater perceived credibility of the treatment. The items were the following:

- - - 1. At this point, how logical does the therapy offered to you seem?
      2. At this point, how successfully do you think this treatment will be able in addressing your symptoms?
      3. How confident would you be in recommending this treatment to a friend who is experiencing similar problems?
      4. By the end of the therapy period, how much improvement in your symptoms do you think will occur?

The treatment credibility scale was added partway through data collection for the study. Ratings were provided by n=21 participants in the RCT Sample (iCBT Coaching n=9; Narrative Coaching n=12). Summed scores were analyzed using an independent samples t-test. There was not a significant difference on credibility ratings between the iCBT Coaching (M=23.11, SD=6.39) and Narrative Coaching (M=23.75, SD=5.58) groups (t(19)=-.24, *p*=.595).

**Coach Adherence Score Sheet**

Circle or highlight the adherence rating that corresponds to each item using the following anchors. For non-adherent items with reverse scoring, 5 corresponds to a 0, 4 corresponds to a 1, etc.

| **Potential Items** | **Adherence** |
| --- | --- |
| Please rate adherence to the Stories of the Mind intervention according to the following items: | 0 = not demonstrated  1 = rarely / briefly demonstrated  2 = sometimes demonstrated  3 = demonstrated fairly often  4 = demonstrated quite often  5 = consistently demonstrated |
| **Process Skills** | |
| Engaged authentically | 0 1 2 3 4 5 |
| Utilized non-verbal skills | 0 1 2 3 4 5 |
| Asked open-ended questions | 0 1 2 3 4 5 |
| Reflected emotions / reinforced effort | 0 1 2 3 4 5 |
| Summarized content | 0 1 2 3 4 5 |
| Avoided advice | 0 1 2 3 4 5 |
| **Psychoeducation** | |
| Treatment / program / weekly rationale | 0 1 2 3 4 5 |
| **Engagement** | |
| Motivational interviewing for participation in program | N/A 0 1 2 3 4 5 |
| **Session Content** | |
| Troubleshooting access of materials | 0 1 2 3 4 5 |
| Review online content | 0 1 2 3 4 5 |
| Session lasted appropriate length (20-30 minutes) | 0 1 2 3 4 5 |
|  | |
| Discussion of CBT model (thoughts/feelings/behaviors) | N/A 0 1 2 3 4 5 |
| Discussion / practice of specific iCBT content (e.g. behavioral activation, thought monitoring, exposure etc.) | N/A 0 1 2 3 4 5 |
| Homework assignment/review (other than watching assigned video) | N/A 0 1 2 3 4 5 |
| Motivational interviewing for change in behaviors (“change talk”) | N/A 0 1 2 3 4 5 |

**Total Adherence Score** (make sure to reverse score non-adherent items) _______

**Overall % Adherence** (total adherence score/# of total possible applicable items) = __________ %

**Number of non-adherent items greater than 0** _______

**Approximate percentage of session time dedicated to adherent items**________

**Alternative Benchmarking Sample: Excluding n=7 Participants who Declined to Participate in the RCT**

Within the Alternative Benchmarking Sample (n=22), there was a significant main effect of Time, such that individuals showed a decrease in HDRS symptoms during the treatment period (b=-3.01, 95% CI:[-4.92, -1.10], f=.450, Z=-3.09, *p*=.002). Of the n=22 patients, n=6 (27.3%) showed clinically significant change. There was a marginally significant Group x Time interaction (f=.183, ꭓ2(1)=3.62, *p*=.057), such that participants in the RCT Sample showed a marginally faster decrease in HDRS symptoms during the treatment period compared to the Alternative Benchmarking Sample. There was a marginally significant pairwise difference (Z=-1.87, *p*=.061), such that the RCT Sample showed marginally lower symptoms than the Benchmarking Sample at post-treatment (all other *p*’s > .222).
